# Preoperative intestine-to-liver CT ratio: useful predictor of resection in strangulated obstruction

**DOI:** 10.1007/s10140-025-02369-8

**Published:** 2025-07-16

**Authors:** Seiichiro Fujishima, Hironori Tsujimoto, Yoshihisa Yaguchi, Hiroyuki Horiguchi, Keita Kouzu, Yusuke Ishibashi, Yujiro Itazaki, Takafumi Suzuki, Naoyuki Uehata, Risa Kariya, Asuma Ide, Hiroshi Shinmoto, Hideki Ueno

**Affiliations:** 1 Department of Surgery, National Defense Medical College, 3-2, Namiki, Saitama Tokorozawa, 359-8513 Japan; 2 Department of Radiology, National Defense Medical College, Tokorozawa, Saitama Japan

**Keywords:** Strangulated bowel obstruction, Intestinal ischemia, Computed tomography, Computed tomography value, Emergency operation

## Abstract

**Background:**

Prompt diagnosis of strangulated bowel obstruction (SBO) is critical because delayed recognition can lead to life-threatening complications. This study assessed whether the intestinal-to-liver CT attenuation value ratio—a comparison of ischemic bowel-wall enhancement to liver enhancement—can predict the need for intestinal resection in SBO patients.

**Materials and methods:**

We retrospectively analyzed 52 patients who underwent emergency surgery for suspected SBO from 2014 to 2021. Of these, 35 required intestinal resection due to irreversible ischemia (resection group), while 17 did not (no-resection group). Preoperative clinical and imaging findings were compared between groups.

**Results:**

The resection group had a longer time from onset to surgery (*p* = 0.034) and higher leukocyte counts (*p* = 0.037). CT values of the poorly enhanced intestinal wall and the intestinal-to-liver attenuation ratio were significantly lower in the resection group (*p* < 0.0001). Multivariate analysis identified time to surgery (OR 5.08; 95% CI 1.106–23.350; *p* = 0.037) and CT attenuation ratio (OR 15.50; 95% CI 2.622–91.686; *p* = 0.0025) as independent predictors of resection. When stratified by the median ratio cutoff (< 0.40 vs. ≥ 0.40), resection rates were 92% and 44%, respectively (*p* = 0.0001). Additionally, CT attenuation ratio had the diagnostic performance (AUROC 0.886; Youden index 0.736; sensitivity 97.1% and specificity 76.5%.)

**Conclusion:**

An intestinal-to-liver CT attenuation ratio below 0.40 is a strong predictor of intestinal ischemia requiring resection in SBO patients.

**Graphical Abstract:**

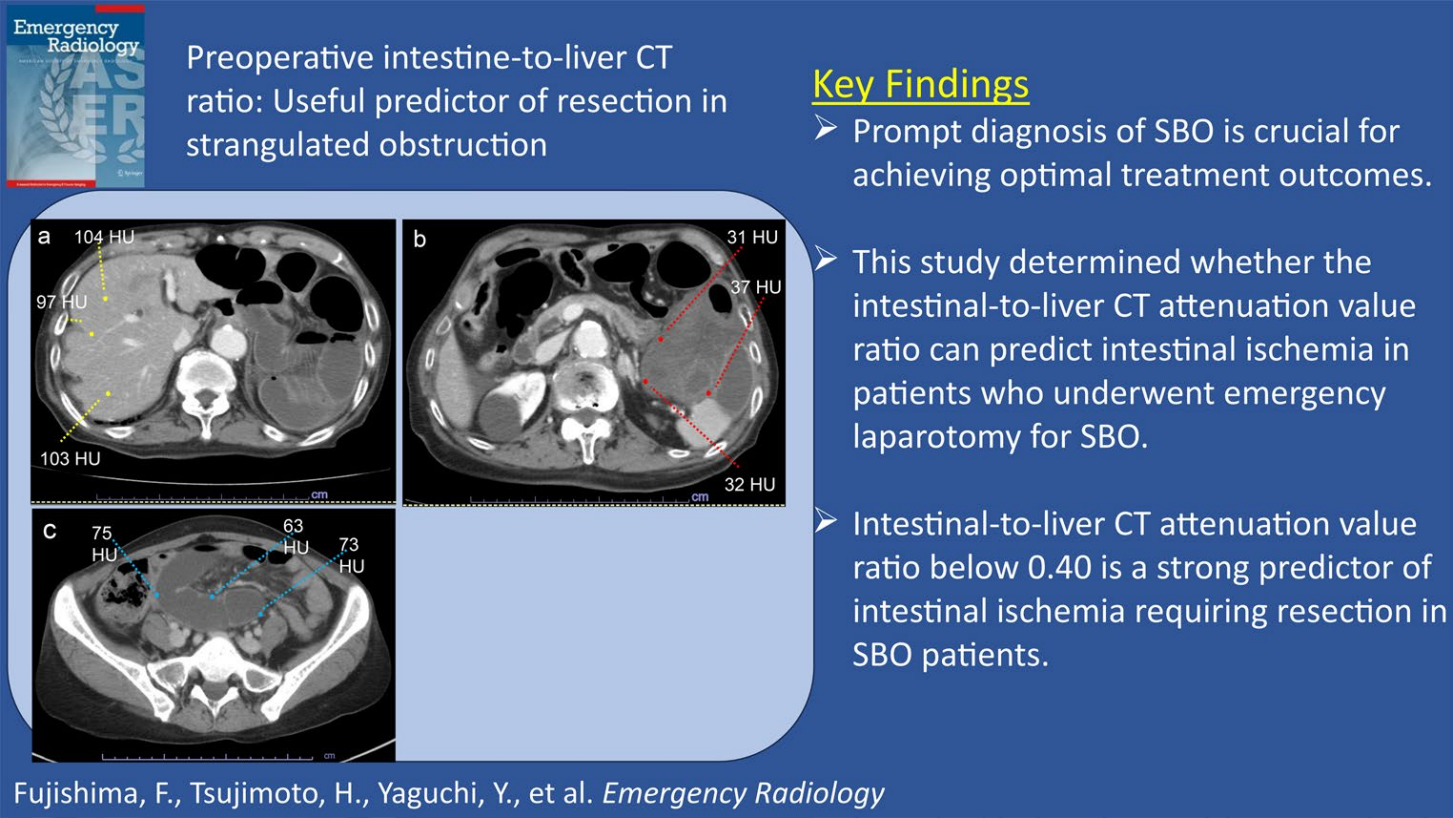

**Supplementary Information:**

The online version contains supplementary material available at 10.1007/s10140-025-02369-8.

## Introduction

Strangulated bowel obstruction (SBO) is a severe acute abdominal condition characterized by intestinal ischemia and requires emergency surgery. SBO can be caused by a fibrous band, volvulus, internal hernia, or adhesions resulting from previous abdominal surgery [1]. Prompt and accurate diagnosis of SBO is crucial for achieving optimal treatment outcomes because it is associated with high mortality rates, ranging from 8 to 25% [2]. The classical clinical signs of SBO include persistent abdominal pain, fever, tachycardia, leukocytosis, peritoneal irritation signs, acidosis, ileus, and hematochezia [3, 4]. However, accurate assessment can be challenging in elderly patients, those regularly taking analgesics, and those with comorbidities [5].

Imaging studies provide crucial information for SBO diagnosis. Computed tomography (CT) can detect SBO with a sensitivity of 73–100% and specificity of 61–100% [6]. Key CT findings include reduced or absent bowel-wall enhancement, ascites, bowel-wall thickening, closed-loop formation, and the whirl sign [7–9]. Wang et al. reported that a CT-based on nomogram that combines radiomic features with clinical data to predict the risk of surgical resection in SBO patients was useful [10]. Additionally, Hirao et al. reported that intestinal fluid CT level was a useful predictor of small bowel ischemia in SBO [11]. Li et al. reported that assessing reduced bowel wall enhancement relative to a proximal dilated bowel loop significantly improved the prediction of bowel necrosis in closed-loop small bowel obstruction [12]. However, there are only a few studies which have been investigated the association between the CT value of poorly enhanced intestinal walls and intestinal ischemia.

This study evaluated the relative attenuation of the CT value of ischemic bowel-wall enhancement to the liver using contrast-enhanced CT and determined whether the intestinal-to-liver CT attenuation ratio can predict intestinal ischemia in patients who underwent emergency laparotomy for SBO. We focused on the liver, which is not closely related to SBO and has uniformly preserved parenchymal tissue, making it suitable as a control.

## Materials and methods

### Patients

This retrospective study enrolled 52 patients who underwent emergency surgery for suspected SBO at our hospital between January 2014 and December 2021. Patients who did not undergo emergency surgery were excluded. We collected the patients’ clinicopathological parameters and surgical procedures from our hospital database and operative records. The diagnosis of suspected SBO was comprehensively determined based on abdominal examination, blood examination, and CT findings. The exclusion criteria were as follows: patients without contrast-enhanced CT and those whose obstruction was caused by a tumor. None of the patients had severe systemic conditions such as shock or advanced liver disease. Among the enrolled patients, 35 patients had irreversible intestinal ischemia and required intestinal resection (resection group) and 17 patients did not require resection (no resection group). We compared the clinical characteristics and CT findings between the two groups.

### CT findings

**Fig. 1:**
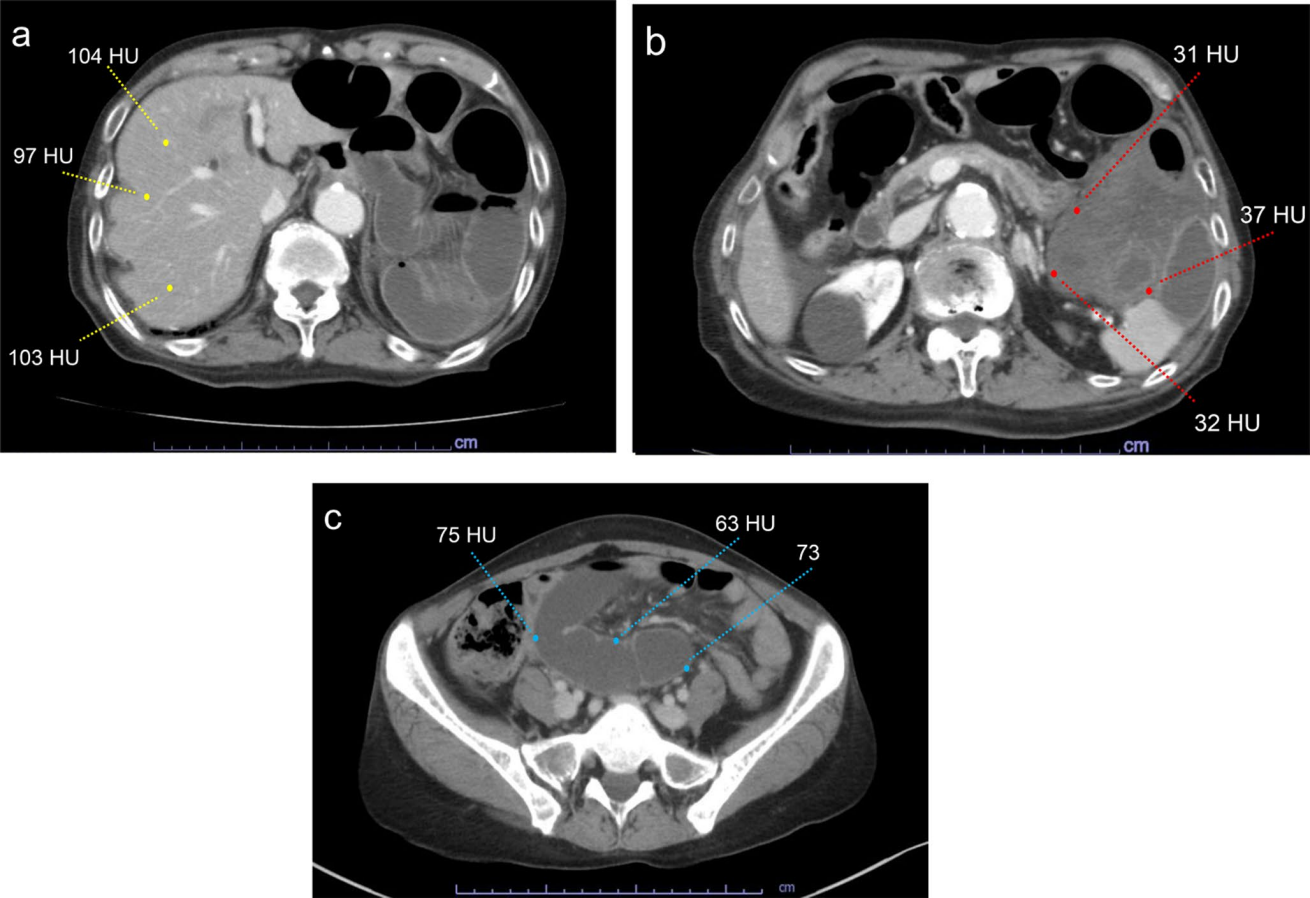
Evaluation of SBO using the CT ratio in the equilibrium phase of the CT images. **a**: An example of randomly selecting three small points of the liver parenchyma. **b**: An example of randomly selecting three small points of the least enhanced intestinal wall. The mean of the CT values was 33.3. This was an example of the CT attenuation ratio below 0.4. **c**: An example of randomly selecting three small points of the least enhanced intestinal wall. The mean of the CT values was 70.3. This was an example of the CT attenuation ratio above 0.4

The patients underwent preoperative abdominal contrast-enhanced CT in both the nonenhanced and equilibrium phases. In our radiology department, CT images were obtained using a 160-detector CT scanner (Aquilion precision; Canon Medical Systems, Tochigi, Japan). CT scans were performed with intravenous contrast material (iohexol Omnipaque 300; GE Healthcare, Japan) administrated at a rate of 1–2 mL/sec by a power injector, based on the patient’s weight. In the equilibrium phase, scans were acquired 120 s after contrast injection. The CT images were reconstructed at a thickness of 5 mm in the axial, sagittal, and coronal planes using an original image with a thickness of 0.5 mm. Two radiologists reviewed the radiographic factors, including the location of poorly enhanced intestinal areas, presence of ascites, closed loop, whirl sign, and presence of mesenteric edema. One of them was a radiologist with 5–7 years of experience and the other was a specialist with more than 8 years of experience. The mean attenuation of the CT values was calculated from three randomly selected small points on the least enhanced intestinal walls and liver parenchyma using axial planes in the equilibrium phase. The intestinal-to-liver CT attenuation ratio was defined as the mean of the intestinal CT attenuation values divided by the mean of the liver parenchyma CT attenuation values (Fig. 1). Two radiologists were blinded to surgical outcomes when they evaluated CT findings and calculated CT attenuation ratio.

### Statistical analyses

Statistical differences between the two groups were evaluated using the Mann–Whitney U test and are expressed as means ± standard deviations (SDs). The associations between factors were analyzed using the chi-square test or Fisher’s exact test. *P*-values < 0.05 were used to denote statistical significance. For the univariate analysis of intestinal resection, clinical and radiographic factors with *p*-values < 0.05 were included in the multivariate analysis. Furthermore, by categorizing the time from onset to surgery, the accuracy, sensitivity, specificity, positive predictive value (PPV), and negative predictive value (NPV) were calculated. The ability to predict intestinal ischemia and intestinal resection was evaluated using the area under the receiver operating characteristic curve (AUROC). All analyses were performed using JMP Pro (version 14.0; SAS Institute Inc., Cary, NC, USA).

## Results

### Clinical and radiographic characteristics of the patients

Table 1 presents the patients’ clinical and radiographic characteristics. A peritoneal irritation sign was observed in 19 patients (36.5%), and 22 patients (42.4%) had preoperative systemic inflammatory response syndrome (SIRS). The causes of SBO were adhesions (*n* = 31, 59.6%), internal hernias (*n* = 14, 26.9%), intestinal volvulus (*n* = 5, 9.6%), and intussusceptions (*n* = 2, 3.9%). CT findings revealed ascites in 51 patients (98.1%), closed loops in 27 patients (51.9%), and the whirl sign in 10 patients (19.2%). The mean CT values of poorly enhanced intestinal walls and liver parenchyma were 41.74 ± 9.31 and 96.60 ± 4.39 HU, respectively. The mean CT values of the liver parenchyma followed a normal distribution. The median intestinal-to-liver CT attenuation ratio was 0.40 (range, 0.29–0.72). Regarding postoperative outcomes, 13 patients (25%) experienced complications, and the mean postoperative hospitalization duration was 11.96 ± 6.91 days. One patient (1.9%) in the resection group died of multiple organ failure.

**Table 1:** Clinical and radiographic characteristics of 52 patients enrolled in this study

	Value
number	52
Age (mean + SD)	69.92 ± 15.18
Gender	
Female	27 (51.9%)
Male	25 (48.1%)
Peritoneal irritation sign	
Present	19 (36.5%)
Absent	33 (63.5%)
Preoperative SIRS	
Present	22 (42.3%)
Absent	30 (57.7%)
Time from onset to surgery (min, median, range)	906 (365–5224)
Cause of SBO	
Adhesions	31 (59.6%)
Hernia	14 (26.9%)
Intestinal torsion	5 (9.6%)
Invagination	2 (3.9%)
Blood test findings	
WBC (/µL, mean ± SD)	12,025 ± 6190.97
CRP (mg/dL, mean ± SD)	2.54 ± 5.88
K (mEq/L, mean ± SD)	4.08 ± 0.69
CK (IU/L, mean ± SD)	180.63 ± 436.84
LDH (IU/L, mean ± SD)	245.27 ± 58.65
Blood gas analysis	
pH (mean ± SD)	7.43 ± 0.09
PaO2 (mmHg, mean ± SD)	95.45 ± 30.58
PaCO2 (mmHg, mean ± SD)	33.65 ± 6.93
Based excess (mEq/L, mean ± SD)	-1.1 ± 4.22
HCO3- (mEq/L, mean ± SD)	22.26 ± 3.72
Lactic acid (mmol/L, mean ± SD)	8.29 ± 12.1
CT findings	
Ascites	
Present	51 (98.1%)
Absent	1 (1.9%)
Closed loop	
Present	27 (51.9%)
Absent	25 (48.1%)
Whirl sign	
Present	10 (19.2%)
Absent	42 (80.8%)
Mesenteric edema	
Present	48 (92.3%)
Absent	4 (7.7%)
CT values of poor contrast intestinal wall (HU, mean ± SD)	41.74 ± 9.31
CT values of liver parenchyma (HU, mean ± SD)	96.60 ± 4.39
CT values ratio (median, range)	0.40 (0.29–0.72)
Postoperative complications	
Present	13 (25.0%)
Absent	39 (75.0%)
Postoperative hospitalization days (mean ± SD)	11.96 ± 6.91
Clinical outcome	
Death	1 (1.9%)
Discharge, Transfer	51 (98.1%)

SD = Standard deviation; SBO = strangulated bowel obstruction; WBC = White blood cell count; CRP = C-reactive protein; CK = Creatine kinase; LDH = Lactate dehydrogenase; CT = Computed tomography

### Comparison of clinical and radiographic factors between the two groups

Table 2 presents the associations between the clinical and radiographic factors. No significant differences in age, sex, peritoneal irritation sign, preoperative SIRS, causes of SBO, CRP, K, CK, LDH, blood gas analysis (pH, PaO_2_, PaCO_2_, HCO^3-^, and lactic acid), or CT findings (ascites, closed loop, whirl sign, and mesenteric edema) were observed between the two groups. However, the resection group had a significantly longer time from onset to surgery (*p* = 0.034) and a higher WBC count (*p* = 0.037) than the no resection group. Furthermore, the CT values of poorly enhanced intestinal walls (*p* < 0.0001) and intestinal-to-liver CT attenuation ratio (*p* < 0.0001) were significantly lower in the resection group than in the no resection group. Regarding postoperative outcomes, no significant differences in the incidence of postoperative complications, postoperative hospitalization, or overall clinical outcomes were observed between the two groups.

**Table 2:** Comparison of clinical and radiographic characteristics between intestinal resection group and no resection group

	Intestinal resection group	No resection group	*P* value
number	35(67.3%)	17(32.7%)	
Age (mean + SD)	69.51 ± 15.10	70.76 ± 15.86	0.784
Gender			
Female	15 (42.8%)	10 (58.8%)	
Male	20 (57.2%)	7 (41.2%)	0.280
Peritoneal irritation sign			
present	15 (42.8%)	4 (23.5%)	
Absent	20 (57.2%)	13 (76.5%)	0.175
Preoperative SIRS			
Present	18 (51.4%)	4 (23.5%)	
Absent	17 (48.6%)	13 (76.5%)	0.056
Time from onset to surgery (min, median, range)	994 (403–5224)	722 (365–2268)	0.034
Cause of SSBO			
Adhesions	21 (60.0%)	10 (58.8%)	
Hernia	8 (22.9%)	6 (35.3%)	
Intestinal torsion	4 (11.4%)	1 (5.9%)	
Invagination	2 (5.7%)	0 (0%)	0.573
Blood test findings			
WBC (/µL, mean ± SD)	13,297 ± 6318	9405 ± 5148	0.037
CRP (mg/dL, mean ± SD)	3.01 ± 6.40	1.58 ± 4.68	0.177
K (mEq/L, mean ± SD)	4.14 ± 0.71	3.95 ± 0.67	0.395
CK (IU/L, mean ± SD)	219.27 ± 526.06	100.94 ± 101.11	0.147
LDH (IU/L, mean ± SD)	256.2 ± 59.42	223.4 ± 52.23	0.136
Blood gas analysis			
pH (mean ± SD)	7.42 ± 0.09	7.46 ± 0.08	0.151
PaO2 (mmHg, mean ± SD)	95.90 ± 33.67	94.42 ± 23.09	0.625
PaCO2 (mmHg, mean ± SD)	34.11 ± 6.13	32.68 ± 8.55	0.741
Based excess (mEq/L, mean ± SD)	-1.35 ± 4.49	-0.55 ± 3.65	0.632
HCO3- (mEq/L, mean ± SD)	22.25 ± 3.52	22.29 ± 4.26	0.755
Lactic acid (mmol/L, mean ± SD)	8.01 ± 13.77	8.75 ± 9.74	0.586
CT findings			
Ascites			
Present	34 (97.1%)	17 (100%)	
Absent	1 (2.9%)	0 (0%)	0.482
Closed loop			
Present	17 (48.6%)	10 (58.8%)	
Absent	18 (51.4%)	7 (41.2%)	0.488
Whirl sign			
Present	6 (17.1%)	4 (23.5%)	
Absent	29 (82.9%)	13 (76.5%)	0.584
Mesenteric edema			
Present	34 (97.1%)	14 (82.4%)	
Absent	1 (2.9%)	3 (17.6%)	0.061
CT values of poor contrast intestinal wall (mean ± SD)	37.03 ± 4.29	51.45 ± 9.40	< 0.0001
CT values of liver parenchyma (mean ± SD)	96.12 ± 4.30	97.59 ± 4.55	0.301
CT values ratio (mean, ± SD)	0.39 ± 0.05	0.53 ± 0.11	< 0.0001
Postoperative complications			
Present	10 (28.6%)	3 (17.7%)	
Absent	25 (71.4%)	14 (82.3%)	0.393
Postoperative hospitalization days (mean ± SD)	12.82 ± 7.14	10.18 ± 6.23	0.084
Clinical outcome			
Death	1 (2.9%)	0 (0%)	
Discharge, Transfer	34 (97.1%)	17 (100%)	0.482

SD = Standard deviation; SBO = strangulated bowel obstruction; WBC = White blood cell count; CRP = C-reactive protein; CK = Creatine kinase; LDH = Lactate dehydrogenase; CT = Computed tomography

### Univariate and multivariate analyses of intestinal resection

Univariate analysis revealed that time from onset to surgery (OR, 3.60; CI, 1.038–12.481; *p* = 0.0435), WBC count (OR, 3.57; CI, 1.062–12.010; *p* = 0.0397), and intestinal-to-liver CT attenuation ratio (OR, 14.375; CI, 2.810–73.527; *p* = 0.0014) were significantly associated with the need for intestinal resection, using the median of time from onset to surgery, the normal upper limit WBC as defined by the Japan Committee for Clinical Laboratory Standards, and the median of CT attenuation ratio as cutoff values. In the multivariate analysis using nominal logistic regression, the time from onset to surgery (OR, 5.08; CI, 1.106–23.350; *p* = 0.037) and intestinal-to-liver CT attenuation ratio (OR, 15.50; CI, 2.622–91.686; *p* = 0.0025) were identified as independent predictive factors (Table 3).

**Table 3:** Predictive factors for intestinal ischemia requiring for SBO by univariate and multivariate analysis

	Univariate analysis		Multivariate analysis	
	OR (95% CI)	*P* value	OR (95% CI)	*P* value
Time from onset to surgery				
< 906	Ref.		Ref.	
≥ 906	3.60 (1.038–12.481)	0.0435	5.08 (1.106–23.350)	0.037
WBC				
< 8600	Ref.		Ref.	
≥ 8600	3.57 (1.062–12.010)	0.0397	2.26 (0.519–9.871)	0.277
CT values ratio				
≥ 0.40	Ref.		Ref.	
< 0.40	14.375 (2.810-73.527)	0.0014	15.50 (2.622–91.686)	0.0025

OR = Odds ratio; WBC = White blood cell count; CT = Computed tomography

### Intestinal resection rate, accuracy, sensitivity, and specificity of the intestinal-to-liver CT Attenuation value ratio (< 0.40) based on time from onset to surgery

**Fig. 2:**
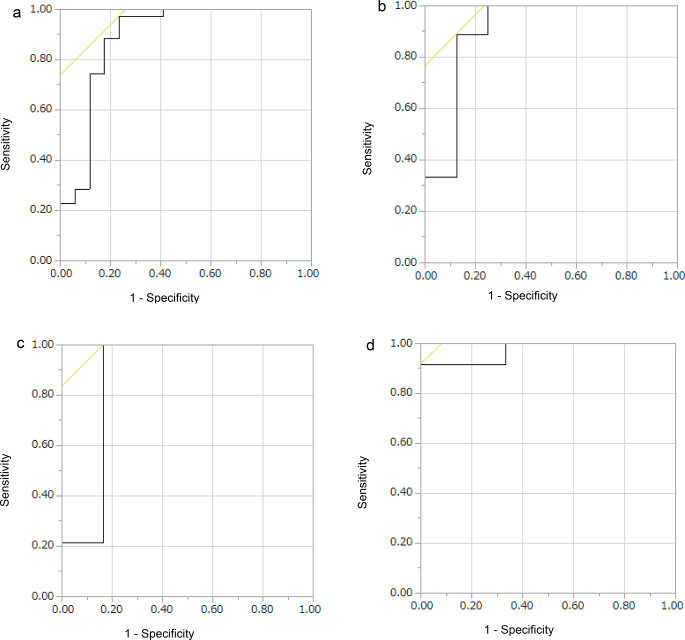
Each ROC curve for categorizing the time from onset to surgery. **a**: ROC curve of the intestine-to-liver CT attenuation ratio for predicting need for resection. AUC was 0.886. **b**: ROC curve within 12 h from onset to surgery. AUC was 0.903. **c**: ROC curve from 12 h to 24 h after onset. AUC was 0.869. **d**: ROC curve from > 24 h after onset. AUC was 0.972

The AUROC of the intestine-to-liver CT attenuation ratio for predicting need for resection was 0.886 (Youden index; 0.736, 95%CI; 0.767–0.957), with an optimal cutoff around 0.47 yielding sensitivity 97.1% and specificity 76.5% (Fig. 2). The intestinal resection rate for intestinal ischemia was evaluated by classifying the median of the intestinal-to-liver CT attenuation ratio as a cutoff value because it was clinically applicable and easy to interpret, and the number of cases is uniformly (Table 4). The resection rate of the CT attenuation ratio (< 0.40) was 92%, and the resection rate of the CT attenuation ratio (≥ 0.40) was 44.4% (*p* = 0.0001). The accuracy, sensitivity, and specificity of the intestinal-to-liver CT attenuation ratio (< 0.40) for predicting the need for intestinal resection within 12 h from onset to surgery were 76.5%, 66.7%, and 87.5%, respectively (Table 5). Furthermore, the PPV and NPV were 85.7% and 70%, respectively. In contrast, for cases in which surgery was performed > 24 h after onset, the accuracy, sensitivity, and specificity were 66.7%, 58.3%, and 100%. The AUROC within 12 h from onset to surgery was 0.903, that from 12 h to 24 h after onset was 0.869, and that from > 24 h after onset was 0.972 (Table 6; Fig. 2).

**Table 4:** Comparison of the resection rate between CT Attenuation ratio ≥ 0.40 and < 0.40

	CT attenuation ratio ≥ 0.40	CT attenuation ratio < 0.40	*P* value
number	27(51.9%)	25(48.8%)	
Intestinal resection			
Yes	12 (44.4%)	23 (92.0%)	
No	15 (55.6%)	2 (8.0%)	0.0001

CT = Computed tomography

**Table 5:** Accuracy, sensitivity, specificity, PPV, and NPV according to the time from onset to surgery for CT values ratio (< 0.40)

Time from onset to surgery	Resection group (*n* = 35)	No resection group (*n* = 17)	Accuracy (%)	Sensitivity (%)	Specificity (%)	PPV (%)	NPV (%)
onset − 12 h	9 (25.7%)	8 (47.1%)	76.5	66.7	87.5	85.7	70
12–24 h	14 (40.0%)	6 (35.3%)	75	71.4	83.3	90.9	55.6
24 h -	12 (34.3%)	3 (17.6%)	66.7	58.3	100	100	37.5

PPV = Positive predictive value; NPV = Negative predictive value

**Table 6:** The area under the ROC curves (AUROC) by categorizing the time from onset to surgery

Time from onset to surgery	AUROC	95% CI	*P* value
onset − 12 h	0.903	0.661–0.991	< 0.0001
12–24 h	0.869	0.644–0.976	0.005
24 h -	0.972	0.737–1.000	< 0.0001

AUROC = area under the receiver operating characteristic curve

## Discussion

In this study, we retrospectively examined the association between intestinal resection and clinical and radiographic characteristics. We demonstrated that the cutoff value of the intestinal-to-liver CT attenuation ratio (< 0.40) was significantly associated with intestinal resection and was an independent predictive factor for intestinal resection in patients with suspected SBO. However, the need for intestinal resection based on the CT attenuation ratio (≥ 0.40) cannot be entirely excluded, given a resection rate of 44.4%. Previous studies have reported that CT is a valuable imaging modality for identifying the cause of intestinal obstruction and determining the presence of intestinal hemodynamic disorders [13, 14]. Various CT signs, such as mesenteric fluid, mesenteric venous congestion, ascites, and reduced bowel enhancement, have been reported as findings associated with strangulated bowel [6, 15–17]. Geffroy et al. highlighted the diagnostic value of increased unenhanced bowel-wall attenuation for detecting strangulation, reporting a sensitivity of 56% and a specificity of 100% in a highly selected population of surgically treated patients [18]. Furthermore, Rondenet et al. reported that increased unenhanced bowel-wall attenuation was the only CT finding significantly associated with bowel necrosis [19]. However, in these reports, gastrointestinal radiologists subjectively assessed the degree of increased unenhanced bowel-wall attenuation, and the evaluation was not quantitative. In clinical practice, evaluating enhancement attenuation is challenging for gastroenterological surgeons and radiologists without quantitative parameters. Therefore, in this study, we focused on CT attenuation values and assessed them objectively and quantitatively. In our methodology, contrast medium was injected into the forearm, and CT scans were acquired 120 s after contrast injection during the equilibrium phase. However, contrast-enhanced CT may be contraindicated in patients with iodine allergy or severe renal dysfunction. Furthermore, CT values of the liver parenchyma were used to adjust for individual variations. In patients with severe systemic conditions such as shock and advanced liver disease, there is a potential risk that the CT value of the liver parenchyma, or both of the liver parenchyma and intestinal walls, could decrease. As a result, false-negative ratio may increase. However, in this study, none of the patients had such severe systemic conditions.

In contrast, as the time from onset to surgery increased, the accuracy, sensitivity, and NPV decreased. Aoki et al. reported that the mechanism underlying the poorly enhanced areas was a relative reduction in the contrast medium, which could be attributed to a decrease inflow, delayed inflow, or an increase in the extracellular water volume [20]. Furthermore, Yamada et al. reported the conditions of SBO as being based on blood flow disorders, categorizing SBO into four phases [21]. Phase 1 was a pre-strangulated state. In phase 1, SBO was caused by adhesions and hernia, among others, no blood flow disorders were observed. Phase 2 was a state due to venous congestion. Dilation of the mesenteric vessels and intestinal edema were noted. Phase 3 was an intestinal ischemic state with a strangulated artery. Phase 4 was an intestinal necrotic state with intestinal necrosis. As the disease progressed through these phases, the CT attenuation values gradually decreased. However, determining the exact phase and duration of SBO based on imaging is challenging. Consequently, the accuracy, sensitivity, and NPV decreased in patients in whom > 24 h had elapsed from onset to surgery. However, the AUROC of the intestinal-to-liver CT attenuation ratio remained high even > 24 h after onset. Therefore, the CT attenuation ratio below 0.40 strongly suggests intestinal ischemia and should prompt immediate surgical intervention, whereas the ratio above 0.40 makes intestinal ischemia less likely, although clinical correlation remains essential.

This study has several limitations. First, this study included a relatively small number of patients and was based on a retrospective, single-institutional design. Second, we did not investigate the interval between CT imaging and surgery. Although all enrolled patients underwent surgery within several hours after CT, the risk of progressive intestinal ischemia remained. Third, inter-observer agreement tests were not performed for the CT ratio measurements by two radiologists, as there were no discrepancies requiring resolution by consensus. Fourth, we did not assess the degree of resected intestinal ischemia *via* pathological diagnosis. Finally, we did not analyze patients who did not undergo emergency surgery. Therefore, multicenter prospective studies with larger sample sizes are warranted to establish a more accurate diagnostic approach for SBO.

## Conclusion

In summary, an intestinal-to-liver CT attenuation ratio below 0.4 was a useful indicator for diagnosing irreversible intestinal ischemia and predicting the need for intestinal resection in patients with suspected SBO, even more than 24 h after onset. This quantitative measure may help identify cases of SBO and guide the urgency of surgical intervention.

## Electronic supplementary material

Below is the link to the electronic supplementary material.

Supplementary Material 1

## Data Availability

All data are available on the request.

## Declarations

### Declare of generative AI in scientific writing

The authors declare no use of AI and AI-assisted technology in the writing process.
