# Supplementary material for: Preoperative intestine-to-liver CT ratio: useful predictor of resection in strangulated obstruction
Source: Emerg Radiol. 2025 Jul 16;32(4):581–9. doi: 10.1007/s10140-025-02369-8 (PMC12328548; doi:10.1007/s10140-025-02369-8)
Supplement: Supplementary file 1 — Supplementary Material 1 [file 10140_2025_2369_MOESM1_ESM.pptx]

## Slide 1
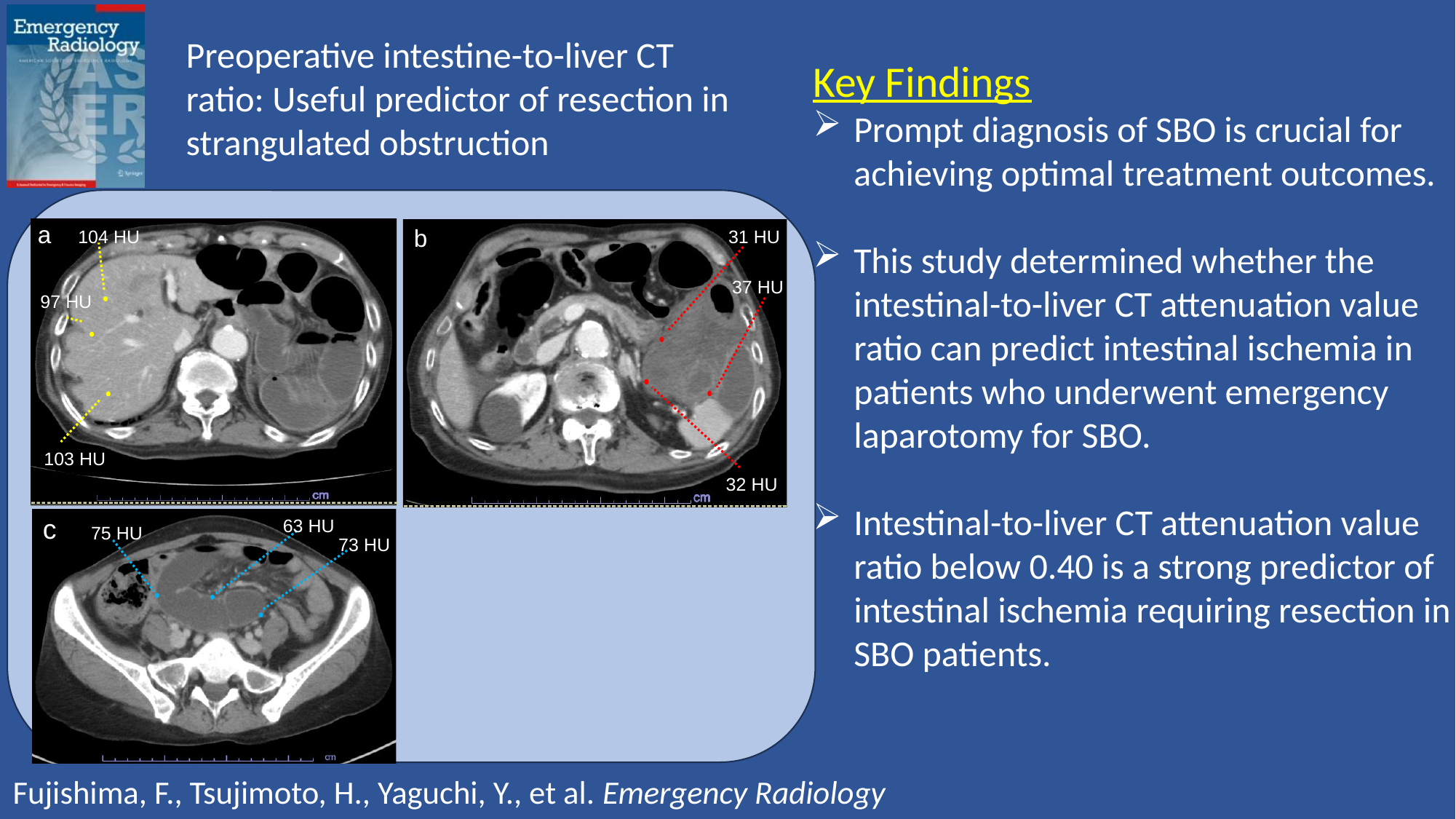

Preoperative intestine-to-liver CT ratio: Useful predictor of resection in strangulated obstruction
Key Findings
Prompt diagnosis of SBO is crucial for achieving optimal treatment outcomes.
This study determined whether the intestinal-to-liver CT attenuation value ratio can predict intestinal ischemia in patients who underwent emergency laparotomy for SBO.
Intestinal-to-liver CT attenuation value ratio below 0.40 is a strong predictor of intestinal ischemia requiring resection in SBO patients.
a
104 HU
97 HU
103 HU
b
31 HU
37 HU
32 HU
c
63 HU
75 HU
73 HU
Fujishima, F., Tsujimoto, H., Yaguchi, Y., et al. Emergency Radiology
